# Glaucoma Surgery in Scleromalacia: Using Endoscopic Cyclophotocoagulation where Conventional Filtration Surgery or Angle Procedures are contraindicated

**DOI:** 10.5005/jp-journals-10028-1227

**Published:** 2017-08-05

**Authors:** Ian AS Rodrigues, Dan Lindfield, Miles R Stanford, Saurabh Goyal

**Affiliations:** 1 Specialist Registrar, Department of Ophthalmology, St Thomas’ Hospital, London United Kingdom; 2 Consultant, Department of Ophthalmology, Royal Surrey County Hospital Surrey, United Kingdom; 3 Professor, Department of Ophthalmology, St Thomas’ Hospital, London United Kingdom; 4 Consultant, Department of Ophthalmology, St Thomas’ Hospital, London United Kingdom

**Keywords:** Minimally invasive glaucoma surgery, Scleromalacia, Secondary angle closure, Secondary glaucoma.

## Abstract

**Aim:**

To describe the surgical management of glaucoma in a patient with severe scleromalacia, and secondary angle closure.

**Introduction:**

The management of glaucoma with coexisting scleromalacia plus secondary angle closure is challenging as most commonly performed incisional glaucoma surgery as well as minimally invasive glaucoma surgery (MIGS), which targets the drainage angle are all contraindicated.

**Case report:**

Medically refractory glaucoma in a 60-year-old male with a 30-year history of granulomatosis with polyangiitis resulting in extensive severe scleromalacia, cicatricial lower lid retraction with significant conjunctival exposure, and widespread synechial angle closure from chronic anterior uveitis was managed with combined phacoemulsification cataract surgery, and endoscopic cyclophotocoagulation (ECP). Careful postoperative management with intensive immunosuppression was used to successfully prevent complications related to the surgery, which resulted in improved visual acuity, and control of intraocular pressure (IOP).

**Conclusion:**

The ECP is a minimally invasive procedure that targets inflow of aqueous, and can be safely and successfully used to control IOP in challenging patients with complex secondary glaucoma, where the use of traditional incisional surgery, and other MIGS procedures are all contraindicated.

**Clinical significance:**

The choice of surgical treatment for medically refractory glaucoma needs to be selected based on the circumstances of individual patients, and take into consideration the condition of the sclera, conjunctiva and drainage angle, against the safety and efficacy of possible treatments.

**How to cite this article:**

Rodrigues IAS, Lindfield D, Stanford MR, Goyal S. Glaucoma Surgery in Scleromalacia: Using Endoscopic Cyclophotocoagulation where Conventional Filtration Surgery or Angle Procedures are contraindicated. J Curr Glaucoma Pract 2017;11(2):73-75.

## INTRODUCTION

There are a number of surgical options for treating medically refractory glaucoma. Traditional incisional surgery, such as trabeculectomy and aqueous shunts (tubes) are commonly used, however, both require normal sclera and conjunctiva to be performed safely. The same is true for other less invasive extraocular procedures, such as trans-scleral cyclodiode laser and deep sclerectomy. In patients with severe scleromalacia, surgical treatment of glaucoma is challenging and limited to intraocular procedures.

## CASE REPORT

A 60-year-old man with a 30-year history of granuloma-tosis with polyangiitis (Wegener’s granulomatosis) was referred with uncontrolled intraocular pressure (IOP) in his left eye. Multiple episodes of severe necrotizing scleritis had occurred in both eyes and resulted in phthisis bulbi with no perception of light of the right eye. Long-term systemic immunosuppression including methotrexate, prednisolone, cyclophosphamide, and rituximab only partially controlled the inflammation in his remaining left eye.

Visual acuity was 6/24 (improving to 6/12 with pinhole) with normal optic nerve appearance and a full visual field. Significant cataract was present. Despite a patent peripheral iridotomy, the peripheral anterior chamber was shallow and gonioscopy documented extensive peripheral anterior synechiae (PAS). This was thought to be a secondary angle closure with the PAS due to chronic inflammation, especially given the axial length was 25.06 mm.

Intraocular pressure was consistently greater than 30 mm Hg despite four IOP lowering agents and oral acetazolamide. Therefore, surgical intervention was considered necessary to control raised IOP of mixed etiology (secondary synechial angle closure, chronic anterior uveitis, and possible steroid induced).

**Fig. 1:**
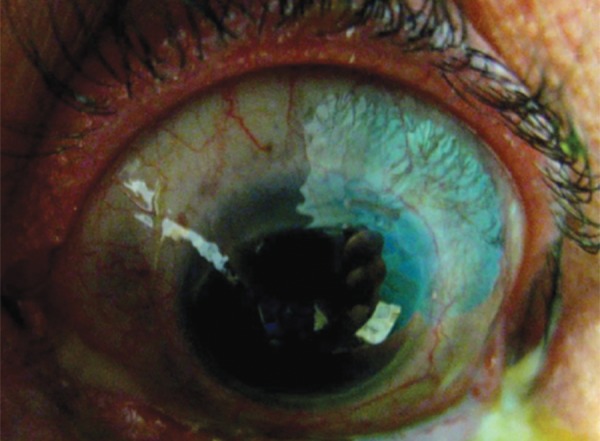
Extensive scleromalacia following recurrent scleritis

**Fig. 2:**
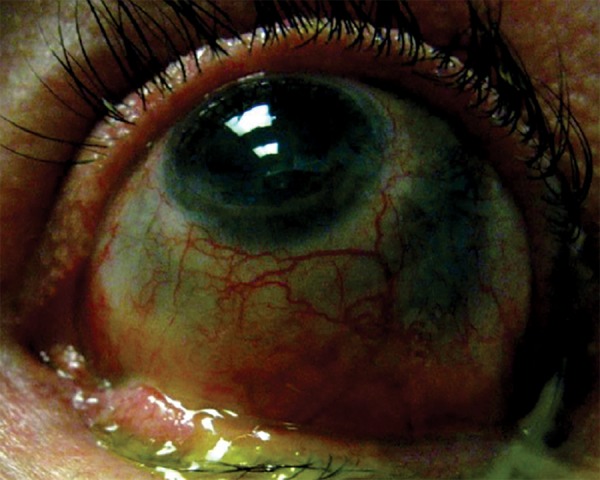
Significant scleral show in upgaze due to cicatricial lower lid retraction

The multiple episodes of scleritis had resulted in extensive severe scleromalacia of the left eye (Fig. 1) with excessive cicatrization of the lower lid causing over 5 mm of inferior scleral show (Fig. 2).

**Figs 3A and B:**
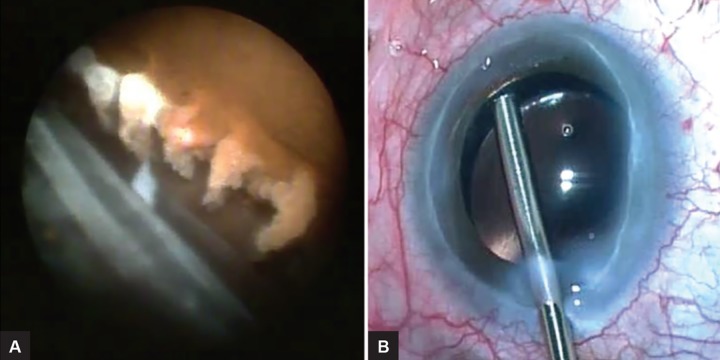
(A) Endoscopic and (B) microscope view of endoscopic cyclophotocoagulation

Choice of glaucoma surgery was very limited with all scleral or trans-scleral modalities and angle-dependent minimally invasive glaucoma surgery (MIGS) contra-indicated due to scleromalacia and extensive synechial closure respectively. Phacoemulsification with endo-scopic cyclophotocoagulation (ECP) was performed (by Goyal) under general anesthesia (Fig. 3). Inflammatory provocation was minimal and the angle, sclera, and conjunctiva were avoided. Pulsed immunosuppressive cover, intracameral dexamethasone, intravitreal triamcinolone acetate (Kenalog), and intensive postoperative preservative free topical steroids were employed to reduce the risk of postoperative inflammation on a background of chronic uveitis.

One year following surgery, IOP remains well-controlled at 12 mm Hg on timolol/dorzolamide combination therapy and vision improved from 6/24 to 6/6.

## DISCUSSION

The surgical management options were both limited and challenging. The extensive scleromalacia in the superotemporal and superonasal quadrants meant placement of a tube or a trabeculectomy was not possible. Inferior tube placement carried unacceptable risk of erosion due to the significant inferior conjunctival exposure and scleromalacia, plus surgically-induced inflammation and scleritis may have led to perforation.

The definition of MIGS tends to encompass any glaucoma procedure that avoids conjunctival dissection and is thus approached via an ab interno clear corneal incision.^1^ Most modalities of MIGS target the drainage angle in an attempt to increase the aqueous outflow, often by using a plethora of permanent intraocular implants.^2^ The presence of widespread PAS meant that performing of any angle-MIGS procedure is relatively contraindicated and would be at high risk of device occlusion.

Endoscopic cyclophotocoagulation is one of the few MIGS procedures that aim to lower IOP by reducing aqueous production.^1^ It has been safely and effectively used for over 20 years.^3^ Endoscopic cyclophotocoagulation also has the benefit of not involving any implant and does not preclude any further glaucoma surgery.^4^

## CONCLUSION

This case illustrates that ECP can be successfully used to control IOP in challenging patients with complex secondary glaucoma where the use of traditional incisional surgery and other MIGS procedures are all contraindi-cated. Endoscopic cyclophotocoagulation is one of few MIGS procedures that reduce aqueous production and is a minimally invasive, safe, and conjunctival-sparing procedure amongst a myriad of MIGS procedures that target the drainage angle to increase trabecular or uveoscleral flow.

## CLINICAL SIGNIFICANCE

The choice amongst the many surgical options for treating medically refractory glaucoma needs to be selected based on the unique circumstance of individual patients.

Factors, such as the condition of the sclera or conjunctiva, the configuration of the drainage angle, and the suitability of permanent intraocular implants need to be considered against the safety and efficacy in context of the level of IOP reduction required of possible treatments.
